# Molecular Profiling of Primary versus Paired Asynchronous Metastatic Clear Cell Renal Cell Carcinoma Reveals Heterogeneity in Tumor Immune Microenvironment

**DOI:** 10.21203/rs.3.rs-7087297/v1

**Published:** 2025-09-25

**Authors:** Brittney Cotta, Srinivas Nallandhighal, Steven Monda, Zayne Knuth, Daniel Triner, Yuping Zhang, Rui Wang, Amy Kasputis, Xuhong Cao, Aaron Udager, Saravana M Dhanasekaran, Ganesh S. Palapattu, Rohit Mehra, Marcin P. Cieslik, Todd M. Morgan, Simpa S. Salami

**Affiliations:** The University of Texas MD Anderson Cancer Center; University of Michigan; University of Michigan; University of Michigan; University of Michigan; University of Michigan; University of Michigan; University of Michigan; Howard Hughes Medical Institute; University of Michigan; University of Michigan; University of Michigan; University of Michigan Rogel Cancer Center; University of Michigan; University of Michigan; University of Michigan

**Keywords:** kidney cancer metastasis, oncologic outcomes, survival, tumor immune microenvironment

## Abstract

**Background and Objective:**

The tumor immune microenvironment (TIME) shows significant heterogeneity in primary clear cell renal cell carcinoma (ccRCC). As TIME heterogeneity between primary and paired metastatic tumors of ccRCC is less understood, we characterize and compare the TIME of primary ccRCC with paired asynchronous metastases.

**Methods:**

We analyzed patients who developed ccRCC recurrence post radical nephrectomy and had both primary and metastatic treatment-naïve tissue available. Capture whole-transcriptome sequencing was performed on formalin-fixed paraffin-embedded (FFPE) specimens using the Illumina platform. Differential gene expression (DGE) analysis and gene set enrichment analysis (GSE) was performed using R packages limma and fgsea respectively. TIME deconvolution was quantified using CIBERSORT, an *in-silico* flow cytometry tool.

**Key Findings and Limitations:**

In aggregate, 42 tumor samples from 19 patients (19 primary tumors with 23 matched metastases) were analyzed. Metastatic sites included lung (n = 6), bone (n = 6), adrenal (n = 4), liver (n = 2), lymph node (n = 2), and soft tissue (n = 3). In unsupervised hierarchical clustering, primary tumors clustered together and not with their matched metastatic tumor. Of the immune cells assayed, primary tumors displayed greater Tregs than their matched (and unmatched) metastases (p < 0.001). Among metastatic sites, bone had high levels of EMT activity compared to their matched primary tumors and lung metastatic tumors were enriched in E2F targets.

**Conclusions and Clinical Implications:**

We demonstrate differences in pathway enrichment and immune cell populations in primary ccRCC and their matched metastases, including a higher infiltration of immunosuppressive T regulatory cells in the tumor immune microenvironment of primary renal ccRCC. Metastatic tumors not only differed from their paired primary tumors but also differed in gene expression, gene set enrichment, and immune cell composition between metastatic tissue sites.

## Introduction

Close to 30% of patients with renal cell carcinoma (RCC) have metastatic disease at time of diagnosis and up to 50% may develop metastases during follow-up or after primary resection.^1^ Historically, patients who developed metastatic RCC had a dismal prognosis with a 5-year survival rate near 10%.^2^ Fortunately, with the advent of targeted therapies such as tyrosine kinase inhibitors (TKIs) and immune checkpoint inhibitors, the outlook of these patients has improved considerably.^3^

However, there remain several unanswered questions for patients with metastatic RCC. First, response rates to immunotherapy at the individual patient level vary, and there are currently no clinically validated genomic biomarkers to predict response.^4^ Second, primary tumors often display a lower responsiveness to immunotherapy than their metastatic sites, with one report demonstrating a 33% partial response rate in the kidney versus a 91% overall response rate in metastatic tumors.^5,6^ Third, the role of the once standard-of-care cytoreductive nephrectomy to reduce tumor burden and improve the effectiveness of systemic therapies remains controversial in the immunotherapy era^7^. Many of these questions remain unanswered as we have yet to fully characterize the complex tumor immune microenvironment of metastatic RCC.

Extensive work from the TRACERx Renal Cohort has identified heterogeneity in driver mutations and copy number variations within not only primary tumors but also matched primary and metastatic tumors.^8,9^ However, fewer studies have assessed the heterogeneity between matched primary and metastatic ccRCC in regard to their immune cell populations. Here, we explored the molecular differences in the tumor immune microenvironment of clear cell renal cell carcinoma by comparing whole transcriptome RNA sequencing of primary renal tumors to their matched metastatic lesions of varying sites.

## Methods

### Patients, samples, and follow-up

Archival samples were collected and experimental protocols approved for genome-based studies by the University of Michigan Institutional Review Board (IRB). All experiments were performed in accordance with relevant guidelines and regulations. Informed consent was waived per the IRB as only de-identified archival samples were used. Patients included those with localized ccRCC (pT1-T3) who underwent both radical nephrectomy and either biopsy or resection of one or more metastatic lesion. Tissue was obtained prior to patients receiving any systemic treatment. Hematoxylin and Eosin (H & E) slides as well as formalin-fixed paraffin-embedded (FFPE) specimens were reviewed by an anatomic pathologist with genitourinary oncology expertise (R.M.) to confirm stage, Fuhrman grade, and identify areas for molecular profiling. Our analyses included comparing pooled primary tumors to pooled metastases samples and comparing matched primary and lung only metastases to matched primary and bone only metastases.

### Capture whole transcriptome sequencing

RNA was isolated from FFPE specimens as previously described^10,11^ using the Qiagen Allprep FFPE DNA/RNA kit (Qiagen, Valencia, CA). Total RNA was quantified using a Nanodrop spectrophotometer and RNA quality was assessed by using Bio-Analyzer. Using 1ug – 6μg of total RNA, exome-capture RNA sequencing was performed as previously described.^12^ Following library preparation, sequencing was performed using the Illumina HiSeq 2500. Briefly, 1 to 6 micrograms of total RNA was used as input in a reverse transcription reaction, followed by second-strand DNA synthesis. Libraries were generated using Sciclone G3 NGS workstation (Perkin Elmer) with the Kappa HT library preparation. Agilent SureSelectXT Human All Exon V5 + lncRNA probes were used for exon capture following the manufacturer’s protocol. Capture transcriptome libraries were analyzed by Agilent 2100 Bioanalyzer for library size and concentration and sequenced by the Illumina HiSeq 2500 (2 × 126-nucleotides read length), with a sequencing coverage of 40–60 million paired reads. Reads that passed the chastity filter of Illumina BaseCall software were used for subsequent analysis.

Following sequencing and base calling, reads were aligned using STAR (2.4.0g1) to the GRCh38.p1 reference genome, using the “basic” version of Gencode 22 to construct the splice junction database. The total number of reads mapping to each locus was counted using feature Counts. TMM function (default settings) on reads mapped to diploid chromosomes were applied to correct for different sequencing depth and effective library size normalization factors. Trimming was not necessary due to the lack of detectable contamination and the soft-clipping capability of STAR. Expression of protein-coding genes was quantified by counting the reads overlapping exons of annotated protein coding genes in strand-specific mode.

### Differential gene expression analyses

We utilized *calcnormfactors* function from R package *edgeR*^13^ to perform library size normalization on counts per million (CPM) data. Samples with library size less than 5 million reads and genes with normalized CPM less than 2 were excluded from the downstream analyses to eliminate samples with poor quality and genes with low expression. Paired differential gene expression (DGE) was performed using R package limma^14^ using the formula: ~Patient.ID + Sample.type. metastatic tissue site information was added to the design matrix as a covariate to adjust for tissue heterogeneity. Genes with Benjamini-Hochberg adjusted p-value < 5% and absolute log-2-fold change > 0.585 (1.5 in linear space) were considered as statistically significant.

### Gene Set Enrichment Analysis

Gene Set Enrichment Analysis (GSEA)^15^ was performed using R package gsva^16^ and fgsea.^17^ We utilized MSigDB^18^ to identify Hallmark gene sets. Benjamini-Hochberg false discovery rates (FDR) < 5% were considered as statistically significant.

### Immune cell deconvolution

Immune cell deconvolution was performed by feeding non-log transformed counts per million (CPM) normalized data as input for CIBERSORT,^19^ an in-silico flow cytometry tool which estimates the proportions of immune cell types based on gene expression data. CIBERSORT has been validated against traditional laboratory-based assays with a high degree of accuracy.^19^ Statistical significance between groups was assessed using the Wilcoxon rank-sum test.

### ESTIMATE

The ESTIMATE^20^ scores, which estimates tumor purity and the presence of stromal and immune cells in tumor tissue, was calculated on non-log transformed CPM normalized data for each tumor sample. Statistical significance between groups was assessed using the Wilcoxon rank-sum test.

### Molecular Cluster Assignments

RNA clusters were assigned to our cohort per methods established on the IMmotion 151 (IMM151) dataset and further validated on the Javelin Renal 101 dataset.^21,22^

We identified shared genes within our dataset and the 10% most variable genes within IMM151 with ultimately 2731 shared genes identified and included. We then trained a random forest machine learning algorithm (R package, randomForest) on the IMM151 data limited to those shared genes. This achieved an out-of-bag error rate of 17.86%. We then used this model on our data to predict IMM151 molecular clusters for each sample. Given the absence of small nucleolar RNA in our dataset, no samples were assigned to cluster 7.

### Survival analysis

We utilized R package survival^23^ to create Kaplan-Meier (KM) curves and multivariable Cox proportional hazard testing to evaluate the independent prognostic impact of the CD8 + and T-regs association on Progression Free Survival (PFS), Disease Specific Survival (DSS) and Overall Survival (OS) by comparing hazard ratios [95% confidence intervals (CI)] and p < 0.05 were considered statistically significant. All downstream data analyses were performed using R statistical software (v 4.1.0, R Core Team 2021).

## Results

### Primary ccRCC exhibits distinct molecular profile compared to matched asynchronous metastases

**Figure 1:**
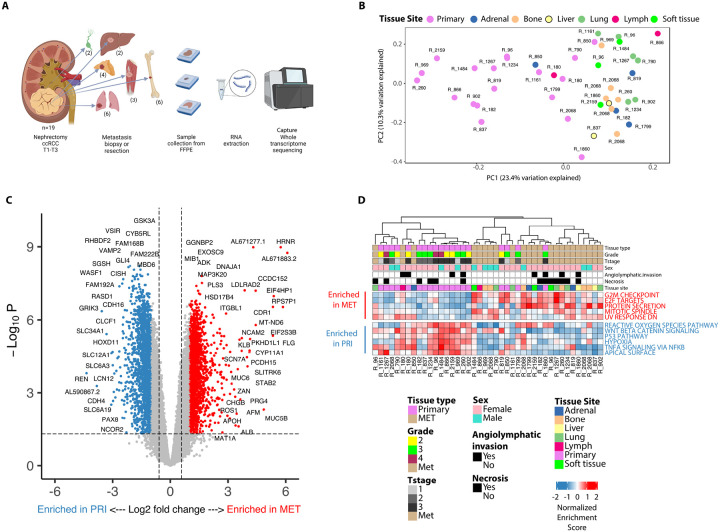
Whole transcriptome analyses of primary and asynchronous metastases in patients with clear cell renal cell carcinoma (ccRCC). **A.** Capture whole transcriptome sequencing was performed on primary nephrectomy specimens from 19 patients and matched asynchronous metastasis specimens from multiple sites comprising Adrenal, Bone, Liver, Lung, Lymph and Soft tissue. **B.** Principal Component Analysis (PCA) plot demonstrating separation of primary and metastatic tumors based on gene expression variation along the PC1 axis. **C.** Volcano plot demonstrating top significantly (FDR p-value < 5%) expressed genes between primary and metastatic ccRCC from differential gene expression (DGE) analysis **D.**MSigDB Cancer Hallmark pathways.

A total of 42 tumor samples from 19 patients (19 primary tumors with 23 matched metastases) were analyzed (Fig. 1A). Metastasis sites included lung (n = 6), bone (n = 6), adrenal (n = 4), liver (n = 2), lymph node (n = 2), and soft tissue (n = 3). All patients underwent radical nephrectomy and 48% had pT3 disease, 52% Fuhrman grade 3, 30% necrosis, 17% angiolymphatic invasion and additional clinical/pathological information available are summarized in Table 1.

We performed capture whole transcriptome sequencing of all primary and metastatic tumors. Principal component analysis (PCA) was performed on 14,971 genes (Fig. 1B) and demonstrated primary tumors clustering together and separate from metastasis of all tissue types, indicating a more similar transcriptomic profile between primary tumors than their matched metastases. DGE analysis of 14,971 genes identified 3,440 upregulated and 3,188 downregulated genes (False Discovery Rate (FDR) < 5%; absolute log_2_ fold-change > 0.585) between primary and metastatic ccRCC tumors and are represented in (Fig. 1C).

We observed substantial heterogeneity in molecular cluster assignments between primary and matched metastases. Among 24 metastases, only 7 were congruent with their matched primary (three in Angiogenic: Cluster 2, two in complement/Omega-oxidation: Cluster 3, one in Angiogenic/Stromal: Cluster 1, and one in Proliferative: Cluster 5). Results of the molecular cluster analysis are demonstrated in supplementary Fig. 2.

### ccRCC metastases harbor genomic features of proliferative disease compared to matched primary tumors

Differential gene expression analyses revealed overexpression of genes linked with aggressive disease in metastases including *DNAJA1* and *HRNR*. Gene Set Enrichment Analysis (GSEA) showed that metastases were enriched in hallmark gene sets associated with proliferative disease biology compared with primary tumors (Fig. 1D). For example, G2M and E2F targets, protein secretion and mitotic spindle were enriched in metastatic tumors compared to primary tumors. The WNT beta catenin, reactive oxygen species, P53, hypoxia and TNFA signaling gene sets were enriched in primary tumors compared to metastases.

### Primary tumors contain a significantly higher proportion of T regulatory cells compared to metastases

We performed immune cell deconvolution using CIBERSORT. The ESTIMATE^20^ scores, which estimates tumor purity and the presence of stromal and immune cells in tumor tissue, was calculated for each tumor type. There were no differences between primary and metastasis sites in stromal, immune, and tumor purity ESTIMATE scores.

**Figure 2:**
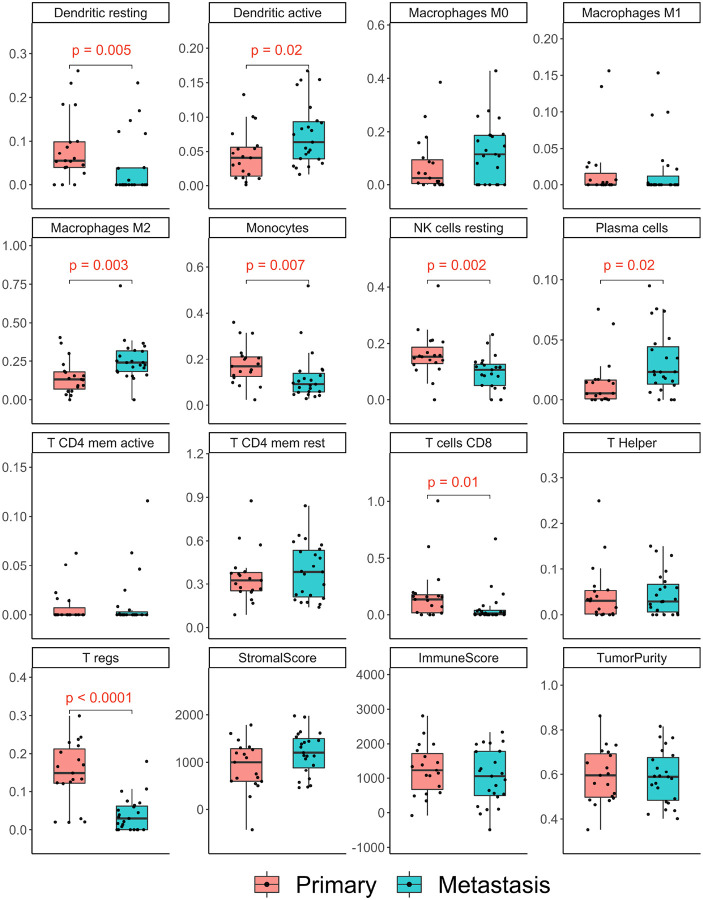
CIBERSORT deconvolution of the tumor immune microenvironment (TIME) differences between primary vs. metastatic ccRCC (all sites). Wilcoxon rank-sum test was used to calculate the p-values displayed.

The immune cell composition differences between primary tumors and metastases from the same tumors are displayed in Fig. 2. Primary tumors displayed a significantly higher proportion of immunosuppressive T regulatory cells (Treg) than metastases (p < 0.0001) and this was consistent across all metastases sites (**Supplementary Fig. 1**). Primary tumors also had greater *resting* dendritic cells, monocytes, resting natural killer (NK), and CD8 + T cells. Metastasis samples displayed higher proportion of M2 macrophages (p = 0.003), with the highest proportion in bone and soft tissue, (**Supplementary Fig. 1**) plasma cells, and *active* dendritic cells.

### ccRCC lung metastases

**Figure 3:**
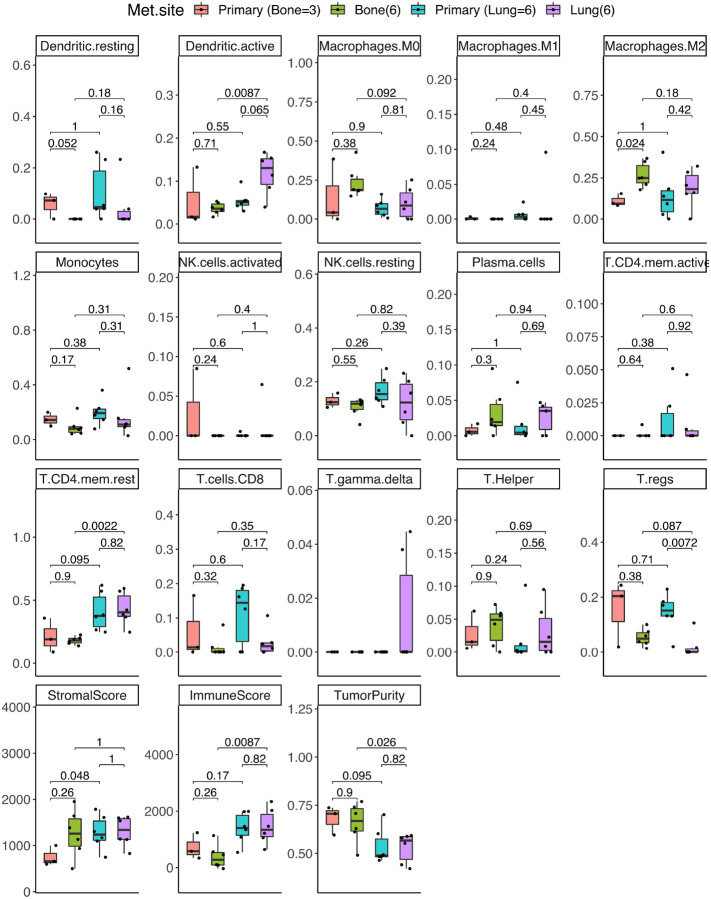
CIBERSORT deconvolution of the tumor immune microenvironment (TIME) differences between primary vs. bone metastases and primary vs. lung metastases. Wilcoxon rank-sum test was used to calculate the p-values displayed.

**Figure 4:**
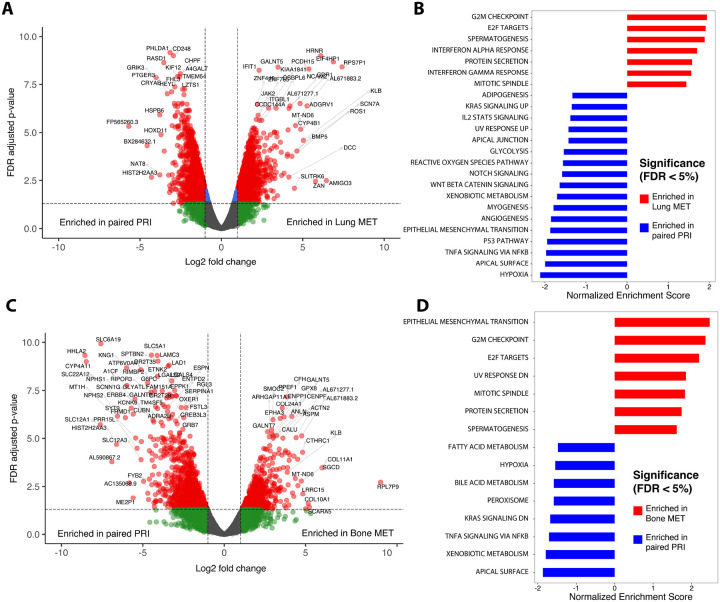
Comparison of the transcriptomic and tumor immune microenvironment (TIME) differences between paired primary ccRCC tumors and bone or lung metastases. **A.** Volcano plot reveals differentially expressed genes between paired primary tumors and lung metastases. Genes to the left are enriched in primary tumors and genes to the right are enriched in lung metastases. **B.** Hallmark pathway analysis demonstrating pathways enriched in either site. **C.** Volcano plot of differentially expressed genes between paired primary tumors and bone metastases. Genes to the left are enriched in primary tumors and genes to the right are enriched in bone metastases. **D.** Hallmark pathway analysis demonstrating gene sets enriched in either site.

We compared lung only (n = 6) metastases to their matched primary tumors using DGE analysis, GSEA and CIBERSORT as described above. Renal primary tumors displayed a significantly greater composition of T regulatory cells than their matched lung metastases (p = 0.0072), (Fig. 3). One of the most significant enriched genes in lung metastases was the gene encoding the protein hornerin (HRNR), which has been identified as an angiogenesis-promoting protein involved in cancer^24^ (Fig. 4A). Hallmark gene sets enriched in lung metastases included the G2M checkpoint and E2F targets (Fig. 4B). The paired primary tumors from patients with lung metastases were enriched in hypoxia gene sets.

### ccRCC bone metastases

We then compared bone (n = 6) metastases to their matched primary tumors using DGE analysis, GSEA and CIBERSORT. Bone metastases displayed greater M2 macrophages than their paired primary tumors (p = 0.024, Fig. 3).

A notable gene enriched in bone metastases is GPX8 (Fig. 4C). The primary renal tumors from which bone metastases occurred displayed high concentration of the gene HHLA2 (Fig. 4C). Epithelial-mesenchymal transition was the most significantly enriched gene set in bone metastases (Fig. 4D). There were no significant differences between primaries of either site (lung or bone) and the primary tumors remained more similar to other renal primaries than their matched metastases, despite metastasizing to different sites (Fig. 3).

### High Treg and low CD8 + T cell infiltration is associated with reduced progression free survival.

**Figure 5:**
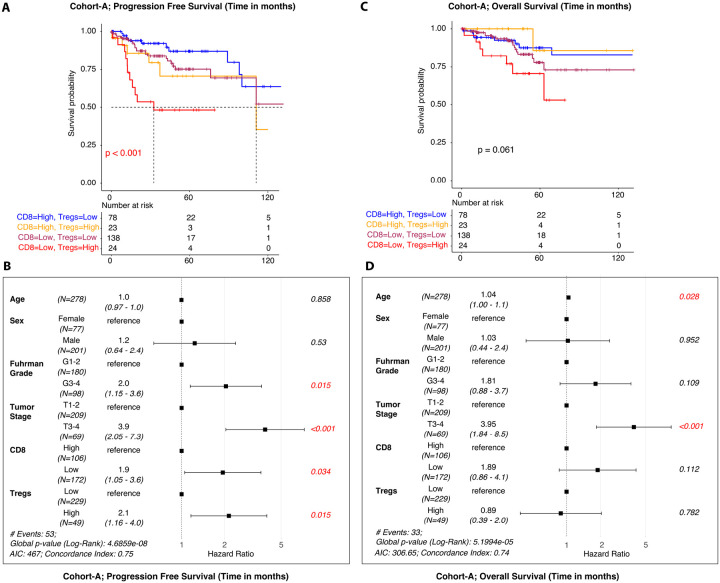
Testing the impact of immune cell infiltration in a combined independent ccRCC datasets (*n = 278*). Two external ccRCC cohorts with oncologic outcomes data were assembled, including: Seishi Ogawa Japanese ccRCC cohort (n=87) and Clinical Proteomics Tumor Analysis Consortium ccRCC cohort (CPTAC; n=191). Upper quartile values (Q3) were used as cut-offs for CIBERSORT scores (CD8 high vs low; Tregs high vs low). **A-B. Progression Free Survival (PFS).** Kaplan-Meier survival curve analysis demonstrated worse PFS in patients with low CD8 and high Treg scores. Multivariable Cox proportional hazard analyses adjusting for clinicopathologic variables showed that low CD8 and high Tregs were independently associated with worse PFS. **C-D. Overall Survival (OS).** Kaplan-Meier survival curve analysis did not demonstrate a significant association with CD8 or Tregs with OS. In multivariable Cox proportional hazard analyses adjusting for clinicopathologic variables, only tumor stage was associated with OS.

We investigated the prognostic impact of CD8 + and T regulatory cell infiltration on ccRCC survival outcomes using a cohort composed of two large publicly available ccRCC gene expression data sets [Seishi Ogawa Japanese ccRCC^25^ (n = 87) and Clinical Proteomics Tumor Analysis Consortium^26^ ccRCC (CPTAC; n = 191)]: Cohort A and The Cancer Genome Atlas (TCGA)^27^: Cohort B. We utilized R package survival^23^ to derive Kaplan-Meier (KM) curves and multivariable Cox proportional hazard testing to validate the independent prognostic impact of CD8 + and T regulatory cell infiltration on progression free survival (PFS) and overall survival (OS). First, we evaluated the prognostic impact of Tregs and CD8 + cells independently by fitting separate Cox proportional hazards models of the form: Surv(time, event) ~ age + sex + grade + stage + cell_type, for each cell type. However, in both cases, the inclusion of either Tregs or T-CD8 cells alone did not yield statistically significant associations with overall survival or progression-free survival. Then, we examined the combined effect of CD8 + and Treg abundances (stratified into low-low, low-high, high-low, and high-high groups). Upper quartile values (Q3) were used as cut-offs for CIBERSORT scores to stratify CD8 + high vs low and Tregs high vs low. In Cohort A, 19% (n = 53) developed recurrence compared with 81% (n = 225) without recurrence after a median follow up of 41 [0, 142] and 42 (0.1, 155] months, respectively. Kaplan-Meier survival analysis demonstrated worse PFS in patients with low CD8 + and high Treg scores and this association was independent of clinicopathologic variables (Fig. 5A–B). However, lower CD8 + or Treg infiltration was not associated with OS (Fig. 5C–D). In cohort B, CD8 + and Treg infiltration were not independently associated with survival outcomes (**Figure S3**).

## Discussion

Characterization of the tumor immune microenvironment is an important and evolving area of research in RCC. Here, we performed whole transcriptome RNA sequencing of a cohort of patients with matched primary and metastatic ccRCC and demonstrate heterogeneity in the immune microenvironment not only between primary and metastatic tumors but also across different sites of metastases. In this cohort, ccRCC metastasis harbor transcriptomic features of aggressive disease compared to matched primary tumors. However, primary tumors displayed a potentially more immunosuppressive microenvironment compared to metastases including high Treg and low CD8 + T cell infiltration which were independently associated with reduced PFS in publicly available genomic datasets. Additionally, we found that significant differences in gene expression, gene set enrichment, molecular clusters, and immune cell composition between metastatic sites from primary ccRCC.

Differential gene expression analyses showed greater expression of genes linked to aggressive disease in our metastasis samples. For example, *DNAJA1*, a member of heat shock protein 40, has been shown to prevent proteasomal degradation of mutant TP53 protein^28^. Tumor suppressor 53 is the most commonly mutated gene in human cancer, and can promote tumor progression through a variety of mechanisms including facilitation of pro-oncogenic tumor microenvironment by altering the secretion of pro-inflammatory cytokines.^29^ Gene HRNR is involved in vascular invasion through angiogenesis and poor tumor differentiation.^30^ It is possible that these genes could influence development of RCC metastases and serve as potential therapeutic targets.

Evasion of antitumor immunity, or immune escape, is purported to be a key driver of primary tumor growth. Thus, our finding of a more immunosuppressive primary tumor microenvironment is consistent with a loss of immunosurveillance that can lead to tumor proliferation. The most striking difference in the tumor microenvironment supporting this finding is the higher concentration of T regulatory cells in primary ccRCC compared to metastases across all tissue types. T regulatory cells play a vital role in the immune system with regard to tolerance to self-antigens, preventing autoimmunity.^31^ This mechanism, however, can similarly lead to tolerance of tumor antigens, suppressing innate antitumor immunity and potentially dampening immunotherapeutic response.^32^ Emerging research also suggests the role of tumor infiltrating Tregs in responsiveness to immune checkpoint inhibitor (ICI) therapy. As Tregs often express CTLA-4 and PD-1 receptors, depletion of immunosuppressive Tregs may account for part of the therapeutic mechanism of these agents.^33^ For example, Romano et al. found that melanoma patients who responded to ipilimumab, an anti-CTLA-4 antibody also utilized in RCC, show decreased intratumoral Treg infiltration after treatment.^34^ Our finding of increased intratumoral Tregs in primary vs metastatic tumors could provide a mechanism for reduced immunotherapy responsiveness in primary ccRCC.

When further evaluating the most common metastatic sites in renal cell carcinoma, lung and bone, we found further deviation from their matched primary tumors. Lung metastases displayed gene set enrichment in the G2M checkpoint and targets of E2F transcription factors. The G2M checkpoint is important for DNA repair and found to be dysregulated in a variety of cancers.^35^ High levels of E2F transcription factors have been associated with tumor aggressiveness, and similarly, in breast cancer, higher levels of E2F associated genes were seen in metastases than in primary tumors.^36^ Renal primary tumors displayed a significantly greater composition of T regulatory cells than their matched lung metastases and bone metastases had greater immunosuppressive M2 macrophages than their matched primary renal tumors. These differences in the immune microenvironment may account for some of the clinical findings in patients with metastases to either site. It has been previously shown that regardless of their primary tumor of origin, lung metastases display a consistently higher immunogenic score.^37^ This is in stark contrast to bone, which tends to be a more immunocompromised microenvironment due to the presence of immature and inhibitory immune cell types.^38^ Clinically, patients with ccRCC metastatic to the bone tend to have worsened cancer outcomes.^39^ In addition, the response of bone metastases to immune checkpoint therapy is also mixed, with one report showing site-specific overall response rates to nivolumab in metastatic RCC of 36% in lung compared to only 5% in bone.^40^ The primary renal tumors from which bone metastases occurred also displayed high concentration of the gene HHLA2. This gene is not generally found in increased concentration in normal kidney tissue.^41^ In cancers, it is a newly discovered immune checkpoint that has both immunostimulant and immunosuppressive functions.^42^ As not all renal cell cancers are responsive to PDL-1 immune checkpoint blockade, the emergence of a new targeted therapy in RCC could be promising. Further insights into the TIME of RCC metastatic to bone may help direct appropriate therapy and improve outcomes for these patients.

Prior studies have demonstrated that a higher Treg and lower CD8 + cell infiltration may prognosticate survival in ccRCC.^43^ Here, we found worse PFS in patients with low CD8 + and high Treg scores and this association was independent of clinicopathologic variables in a novel cohort. Interestingly, survival outcomes were not independently associated with infiltration of Treg or CD8 + infiltration in TCGA. Further research is necessary to determine how further insights into the tumor immune microenvironment of RCC can influence prognostic modeling.

A limitation to our study is inherent in the nature of bulk tumor sequencing, which analyzes a mixture of tumor cells, normal cells, and stromal cells present in a tissue sample. Thus, it can be difficult to distinguish gene expression in normal tissues from changes specific to the tumor. To mitigate this, we added the metastatic tissue site as a co-variate in our analyses. Also, while we hypothesize that differences in the TIME may influence responsiveness to therapy, this study did not contain tumors treated with immunotherapy and this will require further investigation. Despite these limitations, our study has several potential clinical implications. First, separate biopsies of primary and metastatic tumors may be needed to capture the overall disease genomic landscape given tumor heterogeneity. Second, an enriched immunosuppressive T regulatory cell microenvironment in primary ccRCC could provide a biologic rationale for the reduced responsiveness to immunotherapy compared to metastases and a role for cytoreductive nephrectomy. Finally, the heterogenous tumor immune microenvironment across different metastases sites suggests that a multimodal or combinatorial treatment approach may be warranted in metastatic ccRCC.

## Conclusions

Our study provides insights into the heterogeneity between primary ccRCC and their matched metastases, including the observation of an immunosuppressive T regulatory cell-enriched TIME in primary tumors. These findings highlight the need to develop diagnostic tools and treatment paradigms that overcome tumor heterogeneity in metastatic renal cell carcinoma.

## Supplementary Material

Supplementary Files

This is a list of supplementary files associated with this preprint. Click to download.

- finalsuppfigures.pdf

## Figures and Tables

**Table 1:** Clinicopathologic characteristics of the ccRCC patient cohort (n = 19).

Variable		Value (Range/Percent)
Age (years)		62 [50, 77]
Male sex		4 (21%)
Tumor Size (cm)		8 [2.3, 15]
Necrosis		6 (32%)
Angiolymphatic Invasion		4 (21%)
Tumor Purity (in silico estimate)		59% [35, 86]
pT Stage	T1	4 (21%)
	T2	5 (26%)
	T3	10 (53%)
Fuhrman Grade	2	7 (37%)
	3	9 (47%)
	4	3 (16%)
Metastases Sites (Total = 23)	Adrenal	4 (17%)
	Bone	6 (26%)
	Liver	2 (9%)
	Lung	6 (26%)
	Lymph	2 (9%)
	Soft Tissue	3 (13%)

## Data Availability

Data used for downstream analyses such as differentially expressed genes, pathway enrichment scores, Cibersort and Estimate immune deconvolution results are deposited in Gene Expression Omnibus (GEO) under the accession code: GSE278174. R code will be provided by the corresponding author upon reasonable request.
